# Maternal Dietary Inflammatory Status and Serum Neopterin During Pregnancy: Influence on Infantile Atopic Eczema in the Offspring

**DOI:** 10.1002/clt2.70080

**Published:** 2025-07-25

**Authors:** Sarah El‐Heis, Sarah R. Crozier, Evelyn X. Loo, Elizabeth H. Tham, Nicholas C. Harvey, Hazel M. Inskip, Southampton Women's Survey Study Group, Keith M. Godfrey

**Affiliations:** ^1^ Medical Research Council Lifecourse Epidemiology Centre University of Southampton Southampton UK; ^2^ NIHR Southampton Biomedical Research Centre University of Southampton University Hospital Southampton NHS Foundation Trust Southampton UK; ^3^ NIHR Applied Research Collaboration Wessex Southampton Science Park Innovation Centre Southampton UK; ^4^ Singapore Institute for Clinical Sciences (SICS) Agency for Science Technology and Research (A*STAR) Singapore Singapore; ^5^ Department of Paediatrics Yong Loo Lin School of Medicine National University of Singapore Singapore Singapore; ^6^ Khoo Teck Puat—National University Children's Medical Institute National University Health System Singapore Singapore; ^7^ Institute of Developmental Sciences University of Southampton Southampton UK

**Keywords:** infantile atopic eczema, maternal diet, neopterin, proinflammatory diet

## Abstract

**Background:**

A protective influence of maternal inflammatory status on infantile atopic eczema risk has been proposed, but few studies have investigated these potential links. We examined the associations between energy‐adjusted dietary inflammatory index (E‐DII) scores indicative of an inflammatory dietary pattern, maternal serum neopterin levels, a biomarker elevated in Th1 immune activation, and infantile risk of atopic eczema.

**Methods:**

Within the UK Southampton Women's Survey, mothers' diets were recorded using questionnaires at preconception, early and late pregnancy and E‐DII scores derived. 3006 deliveries of live born infants with no major congenital growth abnormalities who were assessed for atopic eczema at 6 or 12 months (ascertained using the UK Working Party Diagnostic Criteria [*n* = 2955 and 2871, respectively]). A sub‐sample of 497 mothers had serum neopterin measured in late pregnancy.

**Results:**

Unadjusted analyses showed that higher E‐DII in preconception and late pregnancy was associated with a lower risk of eczema at ages 6 and 12 months. After adjusting for maternal BMI, age, parity, education, smoking during pregnancy, breastfeeding duration and sex, higher E‐DII in late pregnancy was associated with reduced risks of eczema at age 6 and 12 months (OR 0.89 [95% CI 0.81, 0.99], *p* = 0.03 and OR 0.91 [0.82, 1.00], *p* = 0.05, respectively). Consistent with this, higher maternal serum neopterin was associated with a lower risk of eczema at ages 6 months (OR 0.72 [0.51, 1.01], *p* = 0.05) and 12 months (OR 0.71 [0.53, 0.96], *p* = 0.03).

**Conclusion:**

The findings suggest that a pro‐inflammatory maternal diet and an inflammatory maternal environment during pregnancy may protect the developing infant from Th2 driven inflammation and lower the risk of infantile atopic eczema.

**Trial Registration:**

NCT04715945

## 1 Introduction

Atopic eczema is a common inflammatory skin condition, globally affecting 1 in 5 children [1, 2]. It often runs a chronic relapsing and remitting course and can have a significant impact on sleep, growth and development [3]. It can also have a bearing on the families of affected infants as well as the healthcare system [4].

There is increasing evidence for the importance of early life influences on infantile atopic eczema. Maternal diet before and during pregnancy and specific micronutrient status have been previously examined for their potential to modify the developing immune system risk of offspring atopic eczema. Reports of this have been largely inconsistent [5, 6] but evidence for healthy eating in pregnancy for allergy prevention has been well documented [7, 8, 9, 10].

A lower risk of allergic conditions has been reported in infants whose mothers had higher serum levels of the pro‐inflammatory Th1 markers MCP‐1 and neopterin (produced through stimulation of M1 macrophages and monocytes by interferon‐gamma) during pregnancy [11]. It has therefore been hypothesized that a Th1 pro‐inflammatory intrauterine environment may lower the risk of offspring atopic eczema by diminishing offspring Th2 responses [11], but supportive evidence is sparse.

Dietary inflammatory indices have been used to characterize the inflammatory potential of dietary patterns and can provide insights beyond those from individual nutrients. The Dietary Inflammatory Index (DII) is derived based on 45 dietary parameters linked to circulating levels of 6 inflammatory markers (IL‐1b, IL‐4, IL‐6, IL‐10, TNF‐a, C‐reactive protein) [12]. The energy‐adjusted DII (E‐DII) was subsequently designed. It additionally accounts for potential impacts of energy intake on inflammatory potential and has been shown to correlate with DII. The E‐DII allows more precise comparison of inflammatory potential of diets that vary in caloric intake [13].

Within a mother‐offspring cohort, we aimed to examine the roles of an inflammatory maternal dietary pattern and serum neopterin in modifying the infant's risk of atopic eczema.

## 2 Methods

The UK Southampton Women's Survey (SWS) recruited 12,583 women aged 20–34 years between 1998 and 2002, from the community when they were not pregnant; women who became pregnant were followed up through their pregnancy. Information on maternal lifestyle and socioeconomic status were collected at recruitment [14]. E‐DII was derived from maternal dietary data at preconception, early and late pregnancy as described elsewhere [12]. Individual dietary parameters per 1000 kcal intake were compared to a regionally representative dietary national reference database to derive Z‐scores for the individual parameters; these were then converted to dietary parameter‐specific inflammatory effect scores determined by a comprehensive systematic review [12]. In this study, maternal E‐DII scores were generated from 24 out of the 45 DII dietary parameters (Supporting Information S1: Table 1). Energy intake is an integral part of E‐DII rather than a separate parameter and the 24 parameters have been shown to accurately estimate dietary inflammatory potential [15].

The SWS included 3006 deliveries of live born infants with no major congenital growth abnormalities who were assessed for atopic eczema at 6 or 12 months. Case definition was based on the UK Working Party diagnostic criteria for the definition on atopic eczema [16]; individual history of atopy was omitted as a criterion because the infants were too young to have developed established atopic disorders.

Late pregnancy maternal serum neopterin was measured (BEVITAL AS, Norway) in a subsample (*n* = 497) using liquid chromatography–mass spectrometry/mass spectrometry [17]; coefficients of variation were < 12%. Metabolite concentrations of neopterin were transformed using a Fisher‐Yates transformation [18] to allow analysis of relations per standard deviation (SD) change in neopterin. Univariate and multivariate logistic regression analyses (Stata v18.0, Statacorp LP, TX) related maternal E‐DII and neopterin to infant eczema at ages 6 and 12 months.

**FIGURE 1:**
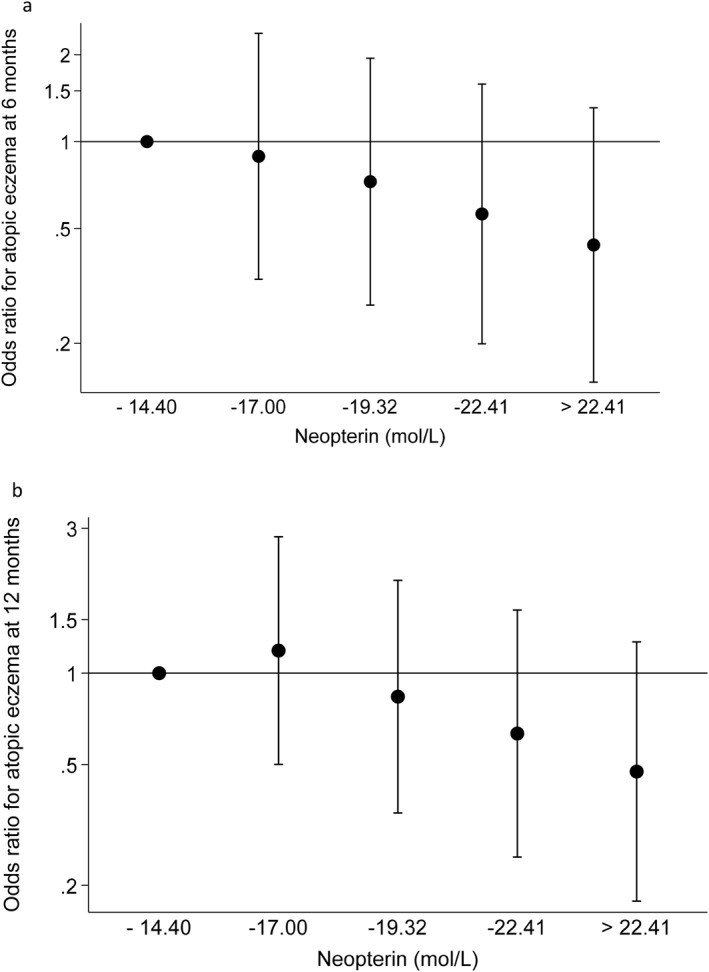
(a) Maternal late pregnancy serum neopterin concentration in relation to offspring atopic eczema at age 6 months (*n* = 392)*. (b) Maternal late pregnancy serum neopterin concentration in relation to offspring atopic eczema at age 12 months (*n* = 372)*. *Adjusted for maternal BMI, education, smoking during pregnancy and history of eczema and infant sex, breastfeeding duration and rs7512552polymorphism.

Directed Acyclic Graphs (DAGs) were used to determine covariates to be included in analyses. In examining the association between E‐DII and infant atopic eczema, maternal education, age, BMI, parity, and smoking during pregnancy were identified as confounders (Supporting Information S1: Figure 1a). A separate DAG examining the association between maternal neopterin and infant atopic eczema identified maternal BMI, education, smoking during pregnancy and eczema in the last 12 months as confounders (Supporting Information S1: Figure 1b). Sex and infant breastfeeding duration were included in all adjusted models to increase the precision of the estimates. A further model additionally adjusting for filaggrin single‐nucleotide polymorphism rs7512552 was designed as this was only available for a subsample (*n* = 1347).

All phases of the Southampton Women's Survey were approved by the Southampton and South West Hampshire Local Research Ethics Committee (329/00,06/Q1702/104) and parents gave written informed consent.

## 3 Results

Table 1 summarizes maternal and infant characteristics. Among the 3006 participants, the mother's average age at child's birth was 30.7 years; 51.5% were primiparous and 15.7% smoked during pregnancy. 51.9% of infants were male; mean infant birthweight was 3.44 kg and gestational age 40.0 weeks. 9.0% and 9.4% had atopic eczema at ages 6 and 12 months, respectively. Supporting Information S1: Table 2 shows that the subgroup of mothers with neopterin measurements in late pregnancy were older at child's birth, smoking was less prevalent and their infants' birthweight was higher.

**TABLE 1:** Characteristics of the study population.

	*n*	Median (IQR), mean (SD) or *n* (%)
Maternal characteristics		
Age at child's birth (y)	3006	30.7 (3.8)
Pre‐pregnancy BMI (kg/m^2^)	2979	24.1 (21.9, 27.4)
A‐level or higher education	2997	1771 (59.1%)
Smoking in pregnancy	2869	451 (15.7%)
Primiparous	3003	1545 (51.5%)
Eczema in the past 12 months	2695	191 (7.1%)
Infant characteristics		
Male	3006	1561 (51.9%)
Gestational age (weeks)	3006	40.0 (39.1, 41.0)
Birthweight (kg)	2979	3.44 (0.55)
Breast feeding (completed months)	2868	
Never breast fed		526 (18.3%)
< 1		581 (20.3%)
1 to 3		615 (21.4%)
4 to 6		484 (16.9%)
7 to 11		421 (14.7%)
12 or more		241 (8.4%)
Filaggrin single‐nucleotide polymorphism rs751255		
C:C	352	26.1%
T:C	643	47.9%
T:T	352	26.1%
6 months assessment		
Age (weeks)	2955	27.4 (26.1, 33.7)
Weight (kg)	2271	7.99 (0.99)
Atopic eczema as per UK working party criteria	2904	262 (9.0%)
12 months assessment		
Age (weeks)	2871	53.7 (52.6, 55.0)
Weight (kg)	2806	10.1 (1.2)
Atopic eczema as per UK working party criteria	270	9.4%

Univariate analysis of E‐DII in relation to infant atopic eczema suggested protective effects of higher E‐DII Scores in preconception and late pregnancy in relation to eczema risk at ages 6 and 12 months (Table 2a). Multivariate analyses for eczema at age 12 months (Table 2b), shows that higher E‐DII scores in late pregnancy were associated with reduced risks of eczema at age 6 and 12 months (OR 0.89 [95% CI 0.81, 0.99], *p* = 0.03 and OR 0.91 [0.82, 1.00], *p* = 0.05, respectively). Early pregnancy E‐DII was not related to atopic eczema at ages 6 and 12 months. Further adjusting for Filaggrin single‐nucleotide polymorphism rs751255 in the subsample had little effect on the odds ratios. E‐DII was not related to maternal late pregnancy serum neopterin concentrations (data not shown).

**TABLE 2:** Univariate (a) and multivariate (b) analysis of the associations between maternal E‐DII scores at preconception, early and late pregnancy and atopic eczema at ages 6 and 12 months.

	Atopic eczema at age 6 months				Atopic eczema at age 12 months			
	*n*	OR	95% CI	*p* value	*n*	OR	95% CI	*p* value
(a) Univariate analysis of E‐DII scores in relation to eczema at ages 6 and 12 months								
Preconception	2903	0.91	0.84, 0.98	0.02	2865	0.91	0.85, 0.99	0.02
Early pregnancy	2091	1.00	0.91, 1.10	0.95	2032	0.97	0.88, 1.07	0.57
Late pregnancy	2498	0.85	0.78, 0.93	< 0.001	2417	0.89	0.82, 0.98	0.02
(b) Multivariate analysis of E‐DII scores in relation to eczema at ages 6 and 12 months^a^								
Preconception	2677	0.98	0.91, 1.07	0.65	2622	0.95	0.88, 1.03	0.23
Early pregnancy	1975	1.06	0.95, 1.18	0.28	1928	0.99	0.89, 1.10	0.85
Late pregnancy	2387	0.89	0.81, 0.99	0.03	2322	0.91	0.82, 1.00	0.05

^a^ Adjusted for maternal education, age, BMI, parity, smoking during pregnancy, breastfeeding duration and infant sex.

Multivariate analyses of maternal neopterin showed that a higher concentration in late pregnancy was associated with a lower risk of atopic eczema at 6 months (OR 0.72 [0.51, 1.01] per SD, *p* = 0.05) and 12 months (OR 0.71 [0.53, 0.96], *p* = 0.03). For illustrative purposes, Figure 1 shows odds ratios of infant atopic eczema according to fifths of the distribution of maternal neopterin; across the range of concentrations higher maternal serum neopterin was associated with a lower offspring risk of eczema.

## 4 Discussion

We found that a higher maternal E‐DII score in late pregnancy was associated with a reduced risk of atopic eczema during infancy. Consistent with this, a higher maternal serum concentration of neopterin, reflecting Th1‐dependent inflammation in the mother, was associated with a lower risk of infant eczema. Collectively, this supports emerging evidence for a protective effect of maternal Th1 inflammation during pregnancy in relation to the offspring's risk of atopic eczema [11, 19].

The mother's immunological profile varies throughout pregnancy, with the first trimester characterized by a Th1 predominant pattern with production of pro‐inflammatory cytokines such as IL‐1β, IL‐2, IFN‐γ and TNF‐α. Later in pregnancy this pattern changes to a Th2 predominant pattern, with B cell activation and production of anti‐inflammatory cytokines such as IL‐4, IL‐5 and IL‐10, followed by a Th1 pattern becoming predominant again in preparation for delivery of the fetus. The influence of maternal inflammatory profile on the developing fetal immune system remains poorly understood. Maternal inflammatory cytokines (MCP‐1, IL‐10, TNF‐α) in late pregnancy have been shown to correlate with offspring's levels of the same cytokines in cord blood and at age 1 year but not with the offspring's risk of atopic eczema [20, 21]. Other studies, however, have shown a strong correlation between maternal and cord blood IFNγ concentrations [22] and that detectable cord blood concentrations of IFNγ are associated with a lower risk of asthma and atopy at age 6 years, and detectable cord blood TNF‐α with a lower risk of atopy [23]. Collectively, this provides some support for the hypothesis that maternal Th1 inflammation (indicated by TNF‐α and IFNγ Th1 inflammatory cytokines) could lower the risk of atopic disorders in the offspring.

Gut dysbiosis has been previously been linked with allergic diseases [24, 25] and diet is known to be a key influence on gut microbial metabolism, with some of the resultant metabolites having immunomodulatory effects [26, 27]. Maternal diet influences the mother's gut microbiome which can potentially impact the risk of offspring atopic eczema through the transmission of protective bacteria to the offspring or through the modulation of immune responses. Additionally, it has been shown that maternal n‐3 PUFA supplementation can affect DNA methylation of genes encoding Th1 and Th2 cytokines and in turn impact offspring Th1/Th2 balance [28] and possibly their risk of atopic eczema. Inflammatory diet during early pregnancy has also been associated with reduced intake of vitamins and minerals and gut microbiota metabolic dysregulation [27].

Previous dietary studies relating maternal micronutrient status before and during pregnancy to offspring atopic eczema have had largely inconsistent findings [5, 6, 29, 30, 31, 32], but dietary inflammatory potential has not previously been examined. Evidence for the role of an inflammatory diet determined by DII in impacting current wheeze in adult and children has been published [33, 34]. The E‐DII is derived from findings related to six inflammatory markers (IL‐1β, IL‐4, IL‐6, IL‐10, TNF‐α, C‐reactive protein), some of which are associated with a Th1 response (e.g., TNF‐ α) and some with a Th2 response (e.g., IL‐4, IL‐6); this may explain our finding of no association between E‐DII and neopterin, alongside the lower statistical power arising from neopterin measurements only being available in a subsample measured at only one time‐point in late pregnancy. A further limitation is that comprehensive measurements of cytokines and inflammatory markers were not available, so we were not able to unravel the complexities and mechanisms of the inflammatory potential of maternal diets and their impact on the developing fetus, but we would continue to advocate for a healthy diet given its established benefits Strengths of our study include its prospective nature and serial assessments of the E‐DII in relation to infantile atopic eczema starting from pre‐conception, which makes it the first of its kind. The ascertainment of atopic eczema at ages 6 and 12 months was standardized and undertaken by trained research staff who administered a questionnaire and performed a clinical examination. Furthermore, a DAG was used to determine covariates to be included in the analyses. This method provides robust and objective means of identifying confounders in observational studies.

Our study raises the possibility that, by altering fetal immune development, offspring atopic disorders may partly arise from the otherwise beneficial effects of lower maternal inflammation. The findings support emerging evidence for a protective impact of a Th1 predominant intrauterine environment on the risk of infantile atopic eczema, raises the possibility that changes in maternal inflammatory status over recent decades could contribute to the longitudinal trends in infantile eczema incidence and highlights the need for nutritional intervention studies to examine the mechanism further.

## Author Contributions

**Sarah El‐Heis:** conceptualization, investigation, methodology, formal analysis, writing – review and editing, writing – original draft. **Sarah R. Crozier:** formal analysis, writing – review and editing, data curation, methodology, validation. **Evelyn X. Loo:** writing – review and editing, conceptualization. **Elizabeth H. Tham:** writing – review and editing. **Nicholas C. Harvey:** writing – review and editing, resources. **Hazel M. Inskip:** writing – review and editing, resources. **Keith M. Godfrey:** conceptualization, writing – review and editing, resources, supervision.

## Conflicts of Interest

K.M.G. has received reimbursement for speaking at conferences sponsored by companies selling nutritional products. K.M.G. is part of an academic consortium that has received research funding from Bayer, Nestec, BenevolentAI Bio Ltd and Danone, outside the submitted work. N.C.H. reports personal fees, consultancy, lecture fees and honoraria from Alliance for Better Bone Health, AMGEN, MSD, Eli Lilly, Servier, Shire, Radius Health, UCB, Consilient Healthcare and Internis Pharma, outside the submitted work. The other authors have no competing interests.

## Supporting information

Supporting Information S1

## Data Availability

The data that support the findings of this study are available on request from the corresponding author. The data are not publicly available due to privacy or ethical restrictions.
