# Supplementary material for: Maternal Dietary Inflammatory Status and Serum Neopterin During Pregnancy: Influence on Infantile Atopic Eczema in the Offspring
Source: Clin Transl Allergy. 2025 Jul 25;15(7):e70080. doi: 10.1002/clt2.70080 (PMC12410369; doi:10.1002/clt2.70080)
Supplement: Supplementary file 1 — Supporting Information S1 [file CLT2-15-e70080-s001.docx]

Supplementary Material

Supplementary Figure 1a. Maternal EDII and offspring atopic eczema Directed Acyclic Graph (DAG)


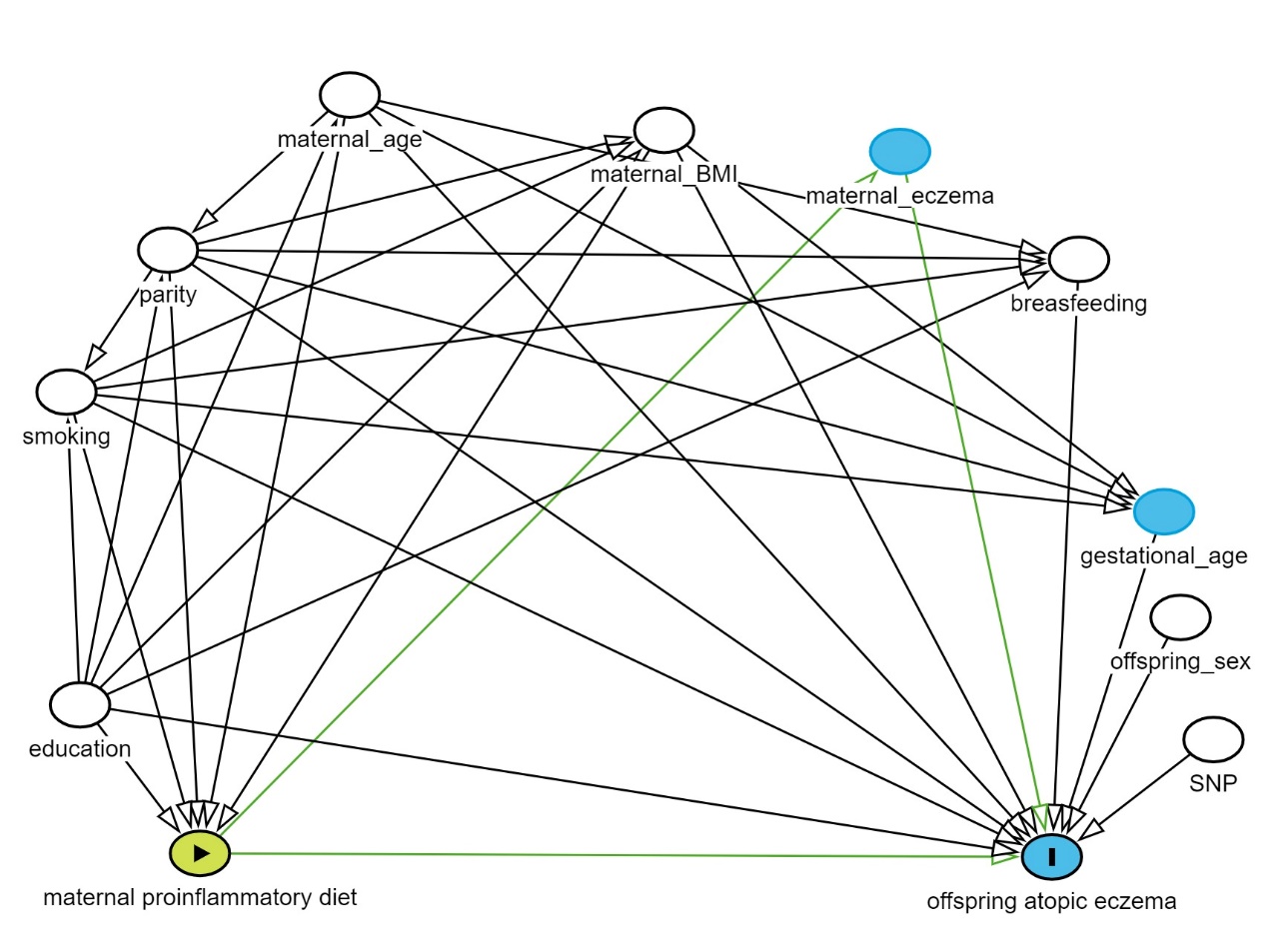


Supplementary Figure 1b. Maternal serum neopterin concentration and offspring atopic eczema Directed Acyclic Graph (DAG)


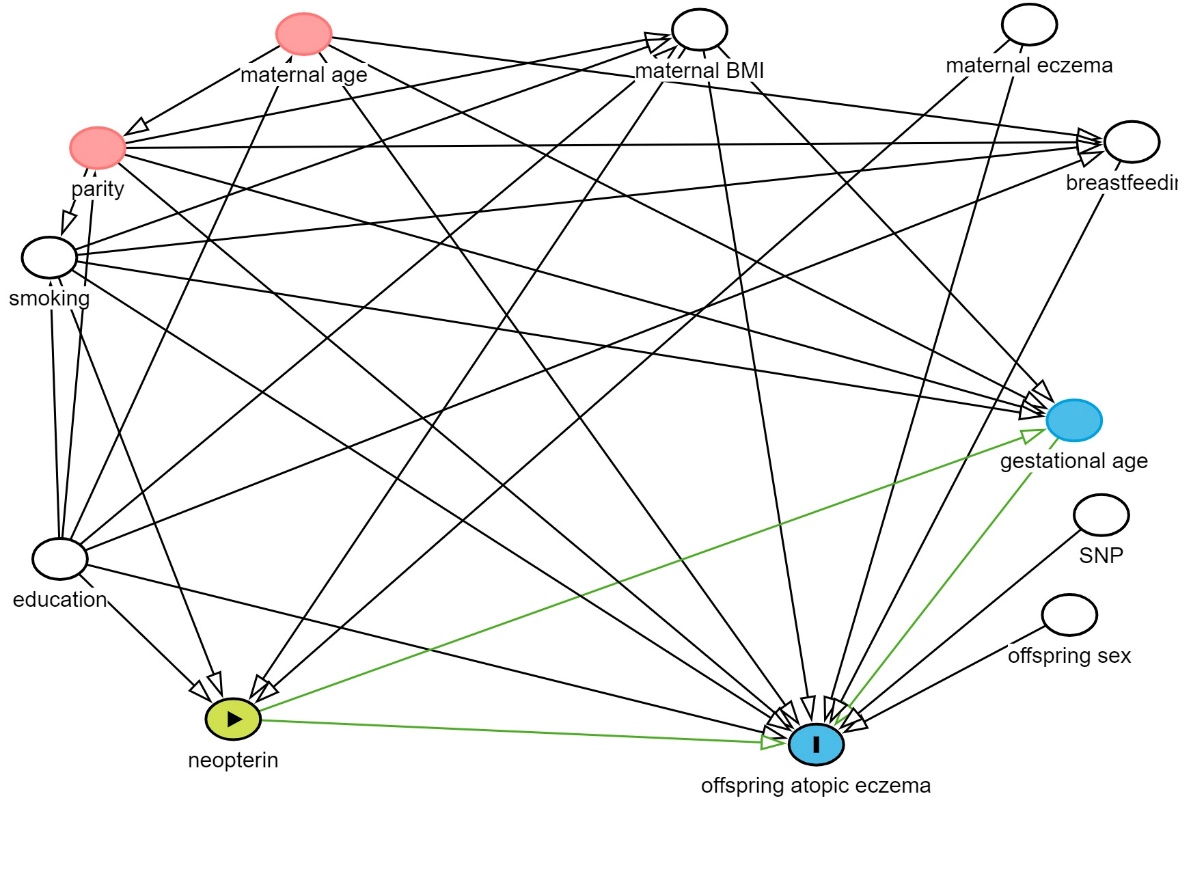


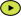
 exposure


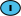
 outcome


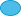
 ancestor of outcome


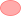
 ancestor of exposure and outcome


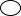
 adjusted variable


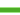
 causal path

Supplementary Table 1. Food parameters included in E-DII generation in SWS

| **Food parameters for E-DII generation** |
| --- |
| Beta Carotene |
| Folic Acid |
| Vitamin A |
| Alcohol |
| Carbohydrate |
| Cholesterol |
| Fat |
| Fiber |
| Iron |
| Magnesium |
| MUFA |
| Niacin |
| Protein |
| PUFA |
| Riboflavin |
| Saturated fat |
| Vitamin B 12 |
| Vitamin B 6 |
| Vitamin C |
| Vitamin D |
| Vitamin E |
| Zinc |
| Onion (in grams) |
| Tea (in grams) |

| Supplementary Table 2. Comparison of the study population with neopterin measurements with the remainder of the SWS participants | | | |
| --- | --- | --- | --- |
|  | With neopterin measurements  (n = 497)  Median (IQR), Mean (SD) or % | Other SWS participants  (n = 2511)  Median (IQR), Mean (SD) or % | P value for difference between the two groups |
| *Maternal* |  |  |  |
| Age at child’s birth(y) | 31.2 (3.5) | 30.6 (3.9) | 0.001 |
| Pre-pregnancy BMI (kg/m^2^) | 24.2 (22.2, 27.2) | 24.1 (21.8, 27.5) | 0.34 |
| % A-level or higher degree | 311 (62.8%) | 1460 (58.4)% | 0.06 |
| % Smoking in pregnancy | 62 (12.5%) | 389 (16.4%) | 0.03 |
| % Primiparous | 254 (51.1%) | 1204 (48.0%) | 0.21 |
|  |  |  |  |
| *Infant* |  |  |  |
| % Male | 252 (50.7%) | 1309 (52.2%) | 0.55 |
| Gestational age (weeks) | 40.1 (39.1, 41.0) | 40.0 (39.0, 41.0) | 0.40 |
| Birthweight (kg) | 3.51 (0.47) | 3.43 (0.56) | 0.003 |
